# Analysis of the chromosomal clustering of *Fusarium-*responsive wheat genes uncovers new players in the defence against head blight disease

**DOI:** 10.1038/s41598-021-86362-4

**Published:** 2021-04-02

**Authors:** Alexandre Perochon, Harriet R. Benbow, Katarzyna Ślęczka-Brady, Keshav B. Malla, Fiona M. Doohan

**Affiliations:** UCD School of Biology and Environmental Science and Earth Institute, College of Science, University College Dublin, Belfield, Dublin 4, Ireland

**Keywords:** Plant breeding, Plant genetics, Plant stress responses, Plant sciences, Agricultural genetics

## Abstract

There is increasing evidence that some functionally related, co-expressed genes cluster within eukaryotic genomes. We present a novel pipeline that delineates such eukaryotic gene clusters. Using this tool for bread wheat, we uncovered 44 clusters of genes that are responsive to the fungal pathogen *Fusarium graminearum*. As expected, these *Fusarium-*responsive gene clusters (FRGCs) included metabolic gene clusters, many of which are associated with disease resistance, but hitherto not described for wheat. However, the majority of the FRGCs are non-metabolic, many of which contain clusters of paralogues, including those implicated in plant disease responses, such as glutathione transferases, MAP kinases, and germin-like proteins. 20 of the FRGCs encode nonhomologous, non-metabolic genes (including defence-related genes). One of these clusters includes the characterised *Fusarium* resistance orphan gene, *TaFROG*. Eight of the FRGCs map within 6 FHB resistance loci. One small QTL on chromosome 7D (4.7 Mb) encodes eight *Fusarium*-responsive genes, five of which are within a FRGC. This study provides a new tool to identify genomic regions enriched in genes responsive to specific traits of interest and applied herein it highlighted gene families, genetic loci and biological pathways of importance in the response of wheat to disease.

## Introduction

Prokaryote genomes encode co-transcribed genes with related functions that cluster together within operons. Clusters of functionally related genes also exist in eukaryote genomes, including fungi, nematodes, mammals and plants^1^. Gene clusters consist of two main classes; paralogous genes that most likely arose by gene duplication, and nonhomologous genes that are physically clustered and co-regulated^2^. The yeast (*Saccharomyces cerevisiae*) genome encodes gene clusters that are important for the use of alternative sources of carbon^3^ and nitrogen^4^. Filamentous fungi contain metabolic gene clusters, especially those involved in secondary metabolic pathways that generate toxins, antibiotics, and other metabolites^5^. In animals, gene clusters play a role in development and defence, such as the major histocompatibility complex (MHC) that encodes diverse proteins involved in adaptive and innate immunity^6^.

Plant gene clusters include paralogous genes and functionally related nonhomologous genes coding for enzymes involved in secondary metabolic pathways^7^. Plant biosynthetic gene clusters produce a variety of secondary metabolites, such as benzoxazinoids, cyanogenic glucoside, polyketides, terpenes and benzylisoquinoline alkaloids^8^. The clustering of genes involved in plant metabolic pathways was first demonstrated in maize (*Zea mays*) for the benzoxazinoid (*Bx*) gene cluster that encodes enzymes involved in the biosynthesis of the cyclic hydroxamic acids involved in defence against microbial pathogens and herbivores^9^. Since then, tens of metabolic gene clusters have been characterised in eudicot and monocot plant species^8^. Paralogous gene clusters are shown to contribute to important agronomic traits such as adaptation to abiotic and biotic stresses^10^; plant disease resistance genes such as lectin receptor kinases (LecRK), wall-associated receptor kinases (WAK) and nucleotide-binding leucine-rich repeat (NLR) containing proteins are often organised into clusters^11–13^. The major quantitative trait locus (QTL) *Submergence-1* (*Sub1*) provides an example of a paralogous plant gene cluster involved in abiotic stress tolerance: it encodes a cluster of ethylene-responsive transcription factors induced after submergence, of which the *Sub1A* allele confers tolerance to prolonged submergence^14^. In tomato, a pathogen-responsive metabolic gene cluster is responsible for falcarindiol biosynthesis, a modified lipid involved in pathogen resistance^15^. In rice, the *Bph3* locus corresponds to a cluster of three *LecRKs* induced during brown planthopper infestation, which function together to confer insect resistance^12^. In wheat, five paralogue salt-responsive cytochrome P450 genes (CYPs) confer salinity tolerance to wheat^16^.

The recently released annotated bread wheat *Triticum aestivum* genome was used as a model. Wheat is a very important crop worldwide with a large and recently annotated genome (RefSeq v1.1), detailing 107,891 high-confidence genes across 21 chromosomes^17^. Fusarium head blight (FHB) is a fungal disease of wheat caused by several *Fusarium* spp., most commonly *Fusarium graminearum*. Resistance to FHB is quantitative, and ~ 500 FHB resistance QTL have been reported in wheat^18^. However, the underpinning genes have only been elucidated for two QTL^19–22^. Additionally, comparative transcriptomics analyses identified other FHB resistance genes but only few have been validated^23^. Consequently, more studies are needed to decipher the molecular mechanisms underpinning wheat-*Fusarium* interactions, to optimise disease resistance breeding. Based on evidence that stress-responsive genes can be clustered in plant genomes, we designed a pipeline to determine if diverse functionally related genes cluster in the genome, using FHB of wheat as a model. Using this pipeline, we captured paralogous and nonhomologous clusters encoding metabolic and non-metabolic genes that were responsive to *F. graminearum* infection of wheat. These *Fusarium*-responsive gene clusters (FRGCs) were assessed for their biological significance using gene co-expression analysis and are discussed with respect to their homology with other plants, their links to defence mechanisms, and their proximity to published FHB QTL.

## Results and discussion

### Identification and characterisation of *Fusarium-*responsive gene clusters across the wheat genome

**Figure 1:**
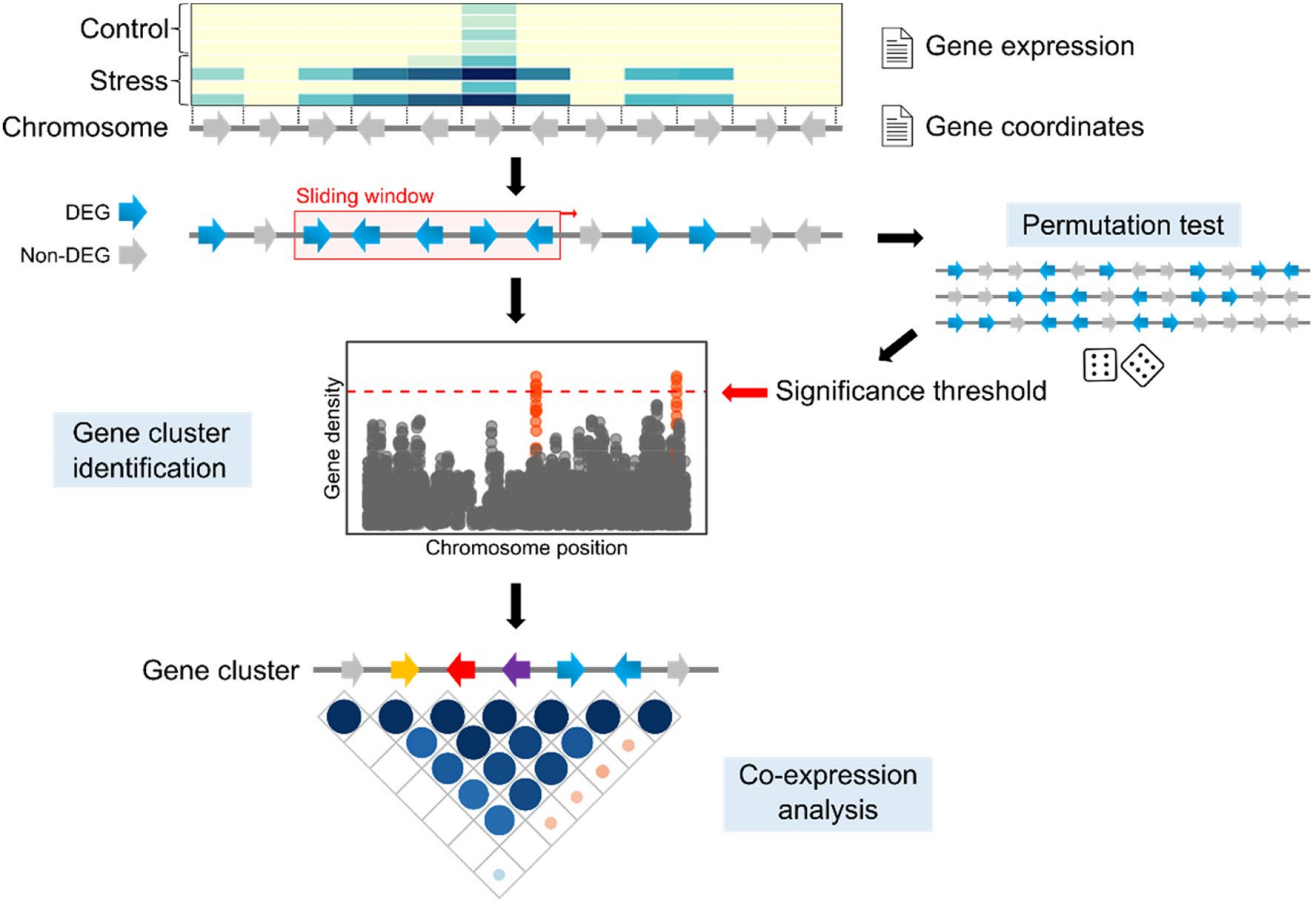
Genome mining of stress-responsive gene clusters. Differential expression analysis was used to identify stress-responsive genes from transcriptomics data. Using chromosomal coordinates of genes from a reference genome, the average number of differentially expressed genes (DEGs) within a sliding window of a user-defined size (hereby called gene density), and the length of a string of consecutive DEGs was calculated. Shuffled data sets were also analysed to determine what values of gene density and consecutiveness can be considered non-random. Clusters of genes that breach this threshold were designated as significantly non-random gene clusters, and their expression profiles are used for co-expression analysis.

We developed a pipeline to determine if wheat genes differentially expressed (DEGs) in response to *F. graminearum* are randomly distributed along the wheat genome, or if they form physical clusters. We used the publicly available transcriptomics dataset from the experiment E-MTAB-4222, wherein heads of wheat near-isogenic lines (NILs) were treated with *F. graminearum* and harvested at 3, 6, 12, 24, 36, and 48 h post inoculation^24^. Differential expression analysis identified 10,567 high confidence (HC) genes that were differentially expressed in response to *F. graminearum* treatment in at least one of the timepoint x NIL combinations (fold change ≥ 1.5; false discovery rate < 0.05). Using these data, a sliding window of 10 genes was used to screen each wheat chromosome and calculate the *Fusarium*-responsive gene (FRG) density and FRG consecutiveness. The sliding window analysis was repeated on 10,000 random permutations of the data to generate a threshold at which density and consecutiveness of DEGs are considered non-random. Using normalised raw expression data for each gene within a cluster, co-expression was measured as the average absolute correlation coefficient between all genes within a cluster and compared to the co-expression of genes outside the cluster. A schematic of the pipeline can be seen in Fig. 1.

**Figure 2:**
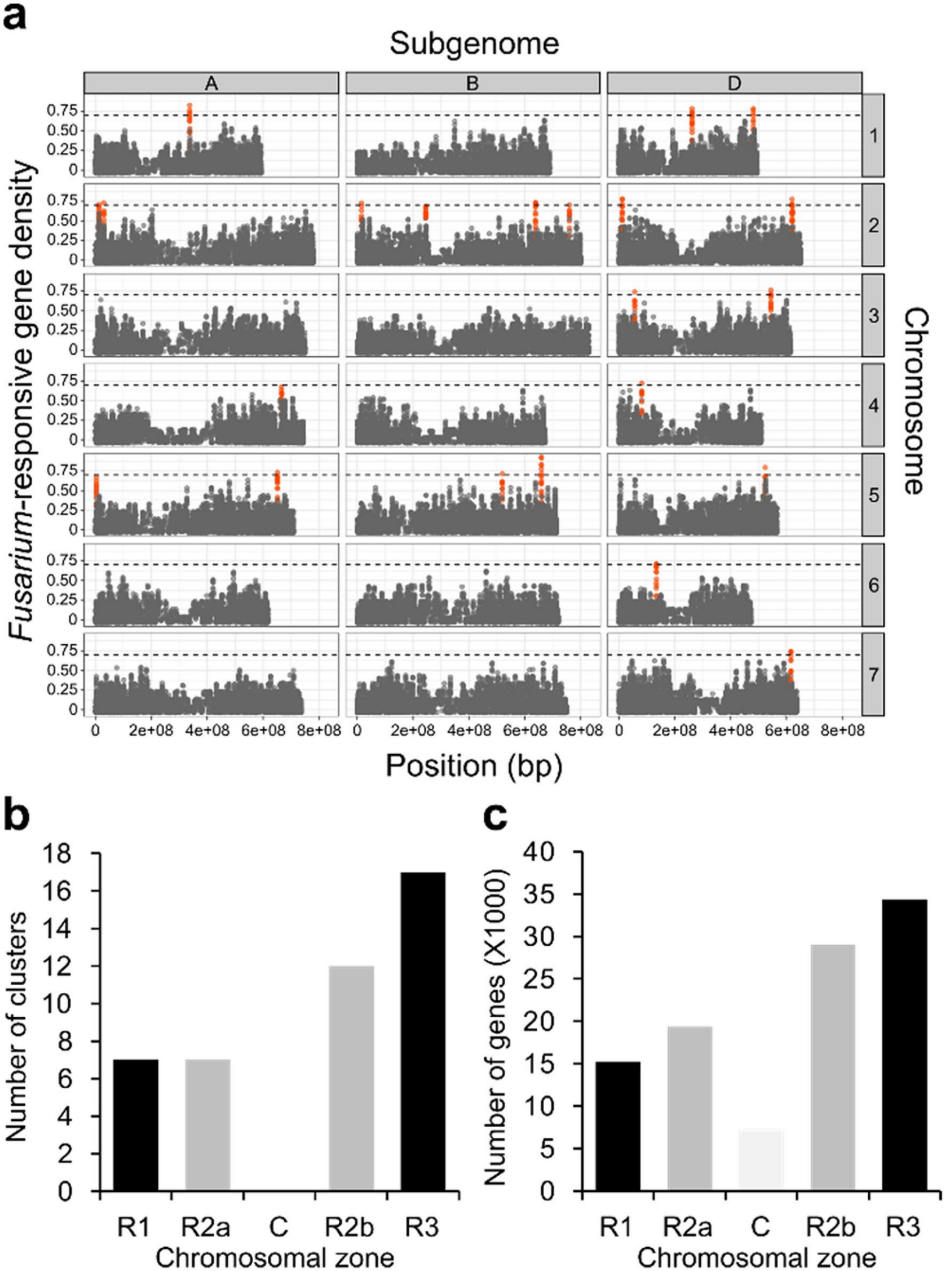
Chromosomal distribution of *Fusarium*-responsive gene clusters (FRGCs). (**a**) *Fusarium*-responsive gene density along each wheat chromosome. A sliding window of 10 genes was used to screen each chromosome and calculate the *Fusarium*-responsive gene density. A significance threshold (dashed line) was calculated by 10,000 random permutations of the data and enriched *Fusarium*-responsive gene loci (those that passed the threshold) were identified and are represented in red vermillion. (**b**) Number of FRGCs and (**c**) total number of high confidence genes are shown in bar diagrams with their distribution in different chromosomal zone such as distal telomeric (short arm (R1) and long arm (R3)), interstitial regions (short arm (R2a) and long arm (R2b)) and centromere regions (C), according to^17^.

In this study, significant clusters were identified as those with a FRG density ≥ 0.7 (Fig. 2a) and/or 5 consecutive FRGs (Supplementary Fig. S1), as these values appeared < 0.01% of the time in the permutation test. We found a total of 44 gene clusters containing 297 FRGs (2.8% of FRGs) located on 16 of the 21 wheat chromosomes (Fig. 2a, Supplementary Fig. S1, Table S1). FRGCs contain between 5 and 11 FRGs, and 5–8 consecutive FRGs, with a physical size between 18 and 1268 kb (Supplementary Table S1), similar to the size of the metabolic gene clusters identified by Schläpfer et al.^25^ (33–284 kb in length, ranging from 4 to 18 genes). The average distance between genes within FRGC is 58 kb, smaller than the average distance between genes near the cluster (10 genes upstream and downstream; 103 kb), or elsewhere in the genome (132 kb), indicating that gene density within FRGCs is high.

### Subgenome distribution, classification and co-regulation of FRGCs

Wheat is hexaploid (AA, BB and DD subgenomes) and the FRGCs were unevenly distributed between the three subgenomes; 12 (27%) were on the A subgenome, 9 (20%) on the B subgenome, 22 (50%) on the D subgenome, and 1 (2%) on an unassigned sequence scaffold (ChrUn). The D subgenome is reported to have an abundance of genes responsive to *F. graminearum*^26^, and we found that it encodes more FRGs than either the A or B subgenomes, both in terms of absolute numbers (3,431, 3,308 and 3670, respectively, on the A, B and D subgenomes) and relative to total gene number per subgenome (9.7, 9.3 and 10.7%, respectively, on the A, B and D subgenomes). D genome enrichment of stress-responsive genes was also noted by Zhang et al.^27^, who identified 143 gene cluster “hotspot regions” in the wheat genome that contain at least 3 consecutive salt-responsive genes. Interestingly, despite the fact that their study used an older genome annotation version and different cut-off criteria for selection of DEGs, 14 of the FRGCs contained the same genes as those identified in the salt-responsive gene clusters (Supplementary Table S1 and Supplementary dataset 3 from Zhang et al.^27^). Thus, some genes contributing to a shared transcriptional response between salt stress and FHB are located within gene clusters.

We determined the chromosomal zone of each FRGC according to the International Wheat Genome Sequencing Consortium (IWGSC)^17^, which separates the chromosomes into three zones: distal telomeric (short arm (R1) and long arm (R3)), interstitial regions (short arm (R2a) and long arm (R2b)), and centromere regions (C). FRGCs were more abundant in telomeric regions compared to interstitial regions (56 and 44%, respectively) and were absent in centromeric region (Fig. 2b). Overall, this distribution correlates with the number of genes present in the different chromosomal zone, with 49,555 genes in the telomeric regions, 48,360 genes in the interstitial regions, and only 7282 genes in the centromeric region (Fig. 2c).

Twenty of the FRGCs have at least one reciprocal homoeologous cluster (Table 1), with 8 pairs and 1 triad. While the homoeologous regions of the remaining 24 FRGCs do contain FRGs, these reciprocal regions did not pass the threshold of density and/or consecutiveness and were therefore not considered FRGC (Table 1 and Supplementary Table S2).

**Table 1:** *Fusarium*-responsive gene cluster categories and contents.

Gene cluster category	Chromosome	Proteins within the cluster	Cluster ID
Nonhomologous, non-metabolic	1A	Embryogenesis-associated protein EMB8, subtilisin-like protease, kinase family protein, transmembrane protein, putative DUF1218 proteins	2
	1B, 1D	Receptor-like kinases, glucan endo-1,3-beta-glucosidase, cysteine-rich receptor kinase	3, 6
	2B	Receptor-like kinases, glucan endo-1,3-beta-glucosidase, receptor kinase, cysteine-rich receptor kinase	12
	2D	Nucleotide-binding leucine-rich repeats, receptor kinase, plastid transcriptionally active 5	16
	2D	B3 domain-containing protein, RING/U-box superfamily proteins, 2-oxoglutarate (2OG) and Fe(II)-dependent oxygenase superfamily protein	17
	2D	Blue copper proteins, FAD-binding Berberine family protein-putative, Ethylene-responsive transcription factor-putative	19
	3D	Lectin receptor kinases, FMN-dependent NADPH-azoreductases, pyruvate decarboxylase, transcription factor, sugar transporter-putative	20
	3D	Glutathione S-transferases, DNAse I-like superfamily protein, SAUR-like auxin-responsive family proteins, nucleotide-binding leucine-rich repeat	21
	4A, 7D	Lectin receptor kinases, cytochrome P450, ABC transporter (Leaf rust resistance gene 34 (LR34)), sugar transporter-putative	23, 40
	4A	Carbonic anhydrases, Coatomer beta subunit, putative, Fatty acyl-CoA reductase, Peroxidase	24
	4D	Fusarium resistance orphan protein (TaFROG), RZ53, lectin receptor kinase, alcohol dehydrogenases, SWAP (Suppressor-of-White-APricot)/surp domain-containing protein, carboxypeptidase	26
	5A, 5B	MYB transcription factor, histone mono-ubiquitination 2, receptor kinase, alcohol dehydrogenases, putative, TSL-kinase interacting protein 1	28, 31
	5D	Kinases putative, cysteine-rich receptor-kinase-like protein, wall-associated receptor kinase-like proteins	35
	6D	Receptor-like kinases, TMV response-related proteins	39
	7D	Protein EXORDIUM-like 1, glycosyltransferase, RNA-binding protein	41
	7D	J-type co-chaperone jac1, mitochondrial, processing peptidase, TMV response-related proteins	42
	7D	Magnesium transporter NIPA, eukaryotic aspartyl protease proteins, 1-amino-cyclopropane-1-carboxylate oxidase 1, DUF668 family protein, chaperone protein dnaJs	43
Nonhomologous, metabolic	2A, 2B, 2D, Un	Cytochrome P450s, kaurene synthases, copalyl diphosphate synthases, UDP-dependent glycosyltransferases, vacuolar sorting receptor family protein, wall-associated receptor kinase-like protein, receptor-like protein kinase	8, 13, 15, 44
	2B	Cytochrome P450s, wall-associated receptor kinase	11
	5A, 5D	Cytochrome P450s, hydroxysteroid dehydrogenases, terpene cyclase/mutases family member, receptor-like protein kinase, always EARLY-like protein, obtusifoliol 14-alpha demethylase	27, 34
	5D	O-methyltransferases, cytochrome P450, chalcone synthases, chalcone-flavonone isomerase family, leucine rich repeat family protein	36
Paralogous, metabolic	1D	**ATP-dependent zinc metalloproteases FtSHs**	7
	2A	**Ribulose bisphosphate carboxylase small chains**, alcohol dehydrogenase, putative	9
	2A	**Phenylalanine ammonia-lyases**, glucuronoxylan 4-O-methyltransferase	10
	5B	**Agmatine coumaroyltransferases**, ornithine decarboxylase	32
Paralogous, non-metabolic	1A, 1D	**Glutathione S-transferases**, RNA binding protein, putative, phospholipase D, alpha/beta-hydrolase superfamily protein	1, 4
	1D	**Mitogen-activated protein kinase kinase kinases**	5
	2B, 2D	**Heavy metal transport/detoxification superfamily proteins**, basic helix-loop-helix (bHLH) DNA-binding superfamily protein, nucleotide-binding leucine-rich repeat	14, 18
	3D	**RZ53s**	22
	4D	**Germin-like protein 1 s**	25
	5A	**Protein of unknown function-DUF538s**, 1-pyrroline-5-carboxylate dehydrogenase	29
	5A, 5B	**Zinc finger proteins**	30, 33
	6A, 6B	**MYB transcription factors**, hexosyltransferase	37, 38

Encoded proteins by *Fusarium*-responsive gene within each cluster are listed. Paralogous proteins that represented ≥ 60% of the total protein content of the cluster are highlighted in bold.

In particular FRGCs 11, 16, 36, and 42 contained many subgenome-specific *Fusarium*-responsive genes, and therefore we concluded they were subgenome-specific FRGCs (Table 1 and Supplementary Table S2).

We categorised FRGCs based on the homology and the metabolic function of their encoded FRGs. FRGCs were classified into four classes on the basis that their encoded FRGs were either paralogous or nonhomologous, and either as metabolic or non-metabolic (Table 1). Most of the clusters were nonhomologous (64%, of which 46% were non-metabolic), and paralogous gene clusters represented 36% of all clusters (of which 27% were non-metabolic) (Table 1). Based on the ‘guilt-by-association’ principle, it is suggested that genes involved in the same biological process are generally co-regulated and co-expressed by a similar regulatory mechanism^28^. Thus, we analysed the correlation between the expression profiles of the FRGs within FRGCs. With the exception of two FRGCs, we found that all clusters had an average Pearson’s correlation coefficient (PCC) ≥|0.3|, and the PCC was significantly higher between the FRGC than the neighbouring genes (*P* ≤ 0.001; Supplementary Fig. S2, Table S3). Half of the clusters had a strong PCC (*r* ≥ 0.7), with 8 clusters having a very strong average correlation between the genes within the cluster (*r* ≥ 0.9). Eleven clusters have a moderate (0.5 ≥ *r* < 0.7) and 7 had a weak (0.3 ≥ *r* < 0.5) correlation.

### Nonhomologous metabolic gene clusters

PlantiSMASH is a bioinformatic tool for secondary metabolic gene cluster (MGC) prediction^29^. We used it to test if the metabolic FRGCs identified were predicted as secondary MGC. We identified 239 MGCs within the wheat genome (Supplementary Dataset 1), of which 10 share common genes with 9 FRGCs, including all 8 of the nonhomologous metabolic FRGCs, with one FRGC matching with 2 different MGCs (Supplementary Dataset 2). From the 10 MGCs identified by PlantiSMASH, 6 encompass entire FRGCs, and include additional genes surrounding the FRGC that, in general, are not responsive to *Fusarium*. Some FRGCs shared some homologies with previously characterised plant metabolic gene clusters, containing strongly co-expressed metabolic genes, and present some link with plant defence mechanisms associated to *Fusarium* diseases. These correspond to a benzoxazinoid-like gene cluster (FRGC 11), momilactone-like gene clusters (FRGC 8, 13, 15 and 44) and terpene metabolic gene clusters (FRGC 27 and 34).

**Figure 3:**
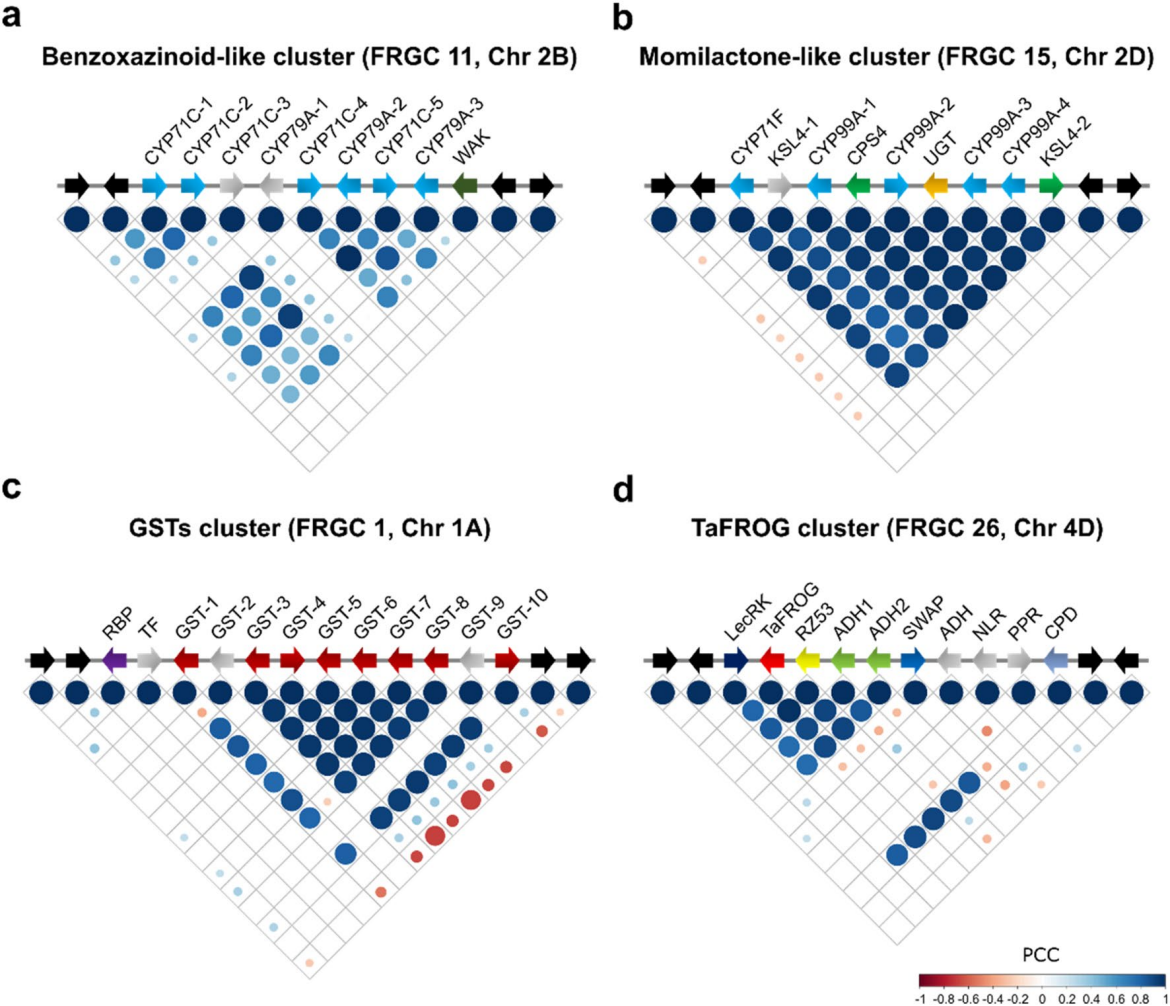
Content, arrangement, and co-expression profiles of representative FRGCs. Schematic representations of *Fusarium*-responsive gene clusters are represented as: *Fusarium*-responsive (coloured arrows), non-responsive genes (grey arrows) and two neighbouring genes (black arrows). Different colours indicate distinct classes of proteins. Co-expression matrices are shown below each cluster. Circle characteristics represent the direction (colour, blue = positive, red = negative) and the strength (size) of the correlation. Four examples of FRGCs are illustrated and correspond to nonhomologous metabolic ((**a**) benzoxazinoid-like and (**b**) momilactone-like cluster), paralogous non-metabolic ((**c**) GSTs cluster), and nonhomologous non-metabolic ((**d**) TaFROG cluster) categories. FRGC ID and chromosome number are indicated between brackets. (**a**) The benzoxazinoid-like cluster contain genes coding for a wall-Associated kinase (WAK) and two class of CYP450s genes with four benzoxazinoid-like genes coding for CYP71C, and two CYP79A genes. (**b**) The momilactone-like cluster is similar to the characterised rice momilactone gene cluster^37^, with CYP450s (CYP) mono‐oxygenases (CYP99A2/A3), two terpene synthase kaurene synthase (KS) and copalyl diphosphate synthase (CPS) genes, but without the momilactone A synthase gene absent in the wheat cluster. (**c**) The GSTs cluster contains an RNA binding protein (RBP) and 8 glutathione-S-transferases (GST) genes. (**d**) The TaFROG cluster contains genes coding for a lectin receptor kinase (LecRK), the *Fusarium* resistant orphan protein (TaFROG), a poaceae-specific protein (RZ53), a SWAP/surp domain-containing protein (SWAP), two alcohol dehydrogenase (ADH) and a carboxypeptidase (CDP). Cluster representation is not to scale.

FRGC 11 is a subgenome specific cluster located on chromosome 2B that shares all FRGs contained within MGC 7 (Supplementary Dataset 2), of which six are cytochrome P450 (*CYP450*) genes and one is a wall-associated kinase (*WAK*) gene (Fig. 3a). Two of the *CYP450*s belong to the *CYP79A* subfamily and four to the *CYP71C* subfamily. The *CYP450s* are strongly co-expressed (*r* = 0.7), while the *WAK* is not (Fig. 3a). Recently, a different *WAK* on chromosome 2AS was proposed to be the gene underlying *Fusarium* resistance *QFhb.mgb-2A*^30^. The CYP79A subfamily is associated with the production of defence molecules, such as cyanogenic glucosides beta‐ and gamma‐hydroxynitrile glucosides and glucosinolates^31^. The CYP71C subfamily proteins are associated with the biosynthesis of the phytoalexin benzoxazinoid (Bx)^32^, and FRGC 11 CYP71Cs are orthologues of a cluster of *CYP71C* genes (36, 56 and 5) on the maize chromosome 2 named “Bx2-like” genes based on their homology to the Bx biosynthetic gene^33^. Benzoxazinoid metabolites have been associated with FHB resistance in Danish wheat varieties^34^ and their degradation by another FHB-causal *species, F. pseudograminearum*, is important for its virulence towards wheat in head blight inoculation assays^35^. Two other wheat *Bx2-like* genes were previously reported to be highly induced by *F. pseudograminearum* infection^36^ but they are not yet functionally characterised and may be involved in alternative secondary metabolic pathways^33^.

Four MGCs predicted by PlantiSMASH match and encompass 4 homoeologous FRGCs (FRGC 8, 13, 15 and 44, Table 1 and Supplementary Table S1) found on chromosome 2A, 2B, 2D and Un, respectively. In these FRGCs, we identified wheat genes coding for core enzymes of a rice momilactone gene cluster^37^, such as the orthologues of the diterpene synthases copalyl diphosphate synthase (*CPS*) *OsCPS4* and kaurene synthase-like (*KSL*) *OsKSL4*, and two *CYP450s* (*CYP99A2*, *3*) genes (as illustrated for FRGC 15 in Fig. 3b). The rice momilactone cluster contains an additional dehydrogenase gene (*OsMAS*; *Os Momilactone A Synthase*)^37^ that is absent in the wheat clusters. Therefore, we named these clusters momilactone-like. The momilactone-like FRGCs contain two additional genes identified as a *CYP71F* and a UDP-dependent glycosyltransferase (*UGT*) for FRGC 8 and 15 (chromosome 2A and 2D), and two *CYP701A* for FRGC 13 (chromosome 2B). All metabolic genes (*CPS*, *KSL*, *CYP99A, CYP71F, CYP701A, UGT*) within the 4 homoeologous FRGCs were induced by *Fusarium* (Supplementary Table S4) and were strongly co-expressed (*r* > 0.85, Supplementary Fig. S2, Table S3), with the FRGs within FRGC 15 showing the strongest correlation (*r* = 0.95, Fig. 3b). Momilactone and related metabolites belong to the diterpenoid phytoalexin group of secondary metabolites, which are involved in antimicrobial activities, allelopathic activities against competing rice weeds, and insect pest antifeedant activities^38^. While rice and maize diterpenoid functions and biosynthesis have been widely studied, key diterpene synthases CPS and KSL remain uncharacterised in wheat^39^. To our knowledge, this is the first time that a diterpenoid gene cluster has been describe in wheat. Momilactone and related diterpenoids were shown to have in vitro activities against fungal pathogens in maize (*F. graminearum, Cochliobolus heterostrophus* and *F. verticillioides*) and in rice (*Xanthomonas oryzae* and *Magnaporthe poae*)^40, 41^. Furthermore, some genetic evidence highlights the role of diterpenoid and diterpene synthases in disease defence. Momilactone gene cluster *OsCPS4* knock-out and knock down rice plants showed enhanced resistance to *X. oryzae* and conversely decreased resistance to the non-host pathogen *M. poae*^42^. Knock-out and knock down of diterpenoid metabolism gene *OsCPS2* increased susceptibility to both *Magnaporthe oryzae* and *X. oryzae*^42^. Maize *ZmKSL2* mutant lines lacking kauralexins were more susceptible to *F. graminearum* stalk rot^41^. Hence, diterpenoid biosynthetic pathways play an important role in plant disease resistance against fungal pathogens, and further studies are needed to examine potential involvement of the wheat momilactone-like clusters in defence mechanisms.

Two homoeologous clusters FRGC 27 & 34 (Table 1) match with terpene metabolic gene clusters (MGC 1 on chromosome 5A and MGC 2 on chromosome 5D, Supplementary Dataset 2). Except for an *F-box* gene included in the MGC, all genes are responsive to *Fusarium*. FRGC 27 & 34 contain three *CYP450s* from the subfamily *CYP51H*, a 3-beta hydroxysteroid dehydrogenase/isomerase, and a terpene cyclase with 85% protein similarity to the rice cycloartenol synthase *OsOSC2*^43^. The CYP51Hs may function in triterpene synthesis, as has been seen for *SAD2*, a CYP51H from *Avena strigosa*^43^*.* Interestingly, *SAD1* codes for a cycloartenol synthase and forms a metabolic cluster with *SAD2*. *SAD2* is involved in the biosynthesis of the avenacin, a triterpenoid saponin that is involved in oat resistance to a variety of fungal pathogens, including the FHB causal agent and DON producer *Fusarium culmorum*^44^. The metabolic cluster FRGC 36 on chromosome 5D matched with MGC 11 and is a polyketide type (Table 1, Supplementary Dataset 2), containing a CYP450 subfamily CYP71C and flavonoid biosynthetic genes coding for four caffeic acid/5-hydroxyferulic acid O-methyltransferases (OMTs), two chalcone synthases (CHSs) and one chalcone isomerase (CHI).

### Paralogous gene clusters

Four of the metabolic and twelve of the non-metabolic FRGCs contain paralogous genes (Table 1). Two of the four metabolic FRGCs contain strongly co-expressed genes (Table S3) and contain genes annotated as ATP-dependent zinc metalloproteases FtSHs (FRGC 7), and phenylalanine ammonia-lyases (*PAL*) (FRGC 10). Ten of the twelve non-metabolic paralogous FRGCs contain strongly co-expressed genes (Table S3). These clusters code for glutathione S-transferases (GSTs) (FRGC 1, 4), Mitogen Activated Protein kinase kinase kinases (MAPKKKs) (FRGC 5), RZ53s (FRGC 22), germin-like proteins (FRGC 25), protein of unknown function-DUF538s (FRGC 29), zinc finger proteins (FRGC 30, 33) and MYBs (FRGC 37, 38).

The *GST* clusters correspond to three homoeologous regions found on the group 1 chromosomes and contain 10 *GST* genes (Supplementary Table S2). Only the *GST* clusters on chromosome 1A (FRGC 1) and 1D (FRGC 4) passed our significance threshold for *Fusarium* response enrichment. As illustrated on Fig. 3c for the chromosome 1A cluster, *GST* clusters are made up with 8 *GSTs* induced for at least two consecutive time points from 12 to 48 h post inoculation with *Fusarium* (Supplementary Table S4) and are highly co-expressed (|PCC|= 0.87–0.92) (Fig. 3c, Supplementary Fig. S2). Non-*GST* genes in both clusters were not, or were slightly, co-expressed with the *GSTs*. *GST* gene clusters are common in plants, and in bread wheat *GSTs* form 37 gene clusters^45^. During plant-pathogen interactions, GSTs can function in diverse biological processes and recently, it has been shown that *Fhb7*, a major FHB resistance gene, encodes for a GST that confers resistance to *Fusarium* by detoxifying trichothecene mycotoxins via glutathione conjugation^22^. While the *Fhb7* GST belongs to the fungal GST etherase-related class^22^, GSTs from FRGC 1 and 4 correspond to the GST class Tau (GSTU)^45^. Interestingly, a *GSTU* cluster in cotton (*Gossypium hirsutum*) that plays an important role in Verticillium wilt resistance^46^, codes for GSTs with high similarity (> 60% identity) to some of the wheat GSTs within FRGC1 and 4. In cotton, over-expression or silencing of GSTs enhanced resistance and susceptibility to *Verticillium dahlia*, respectively^46^. The authors suggested that the cotton GST cluster might enhance plant resistance to *V. dahlia* by fine tuning the balance between production and scavenging of H_2_O_2_.

### Case study: the nonhomologous, non-metabolic *TaFROG* cluster

**Figure 4:**
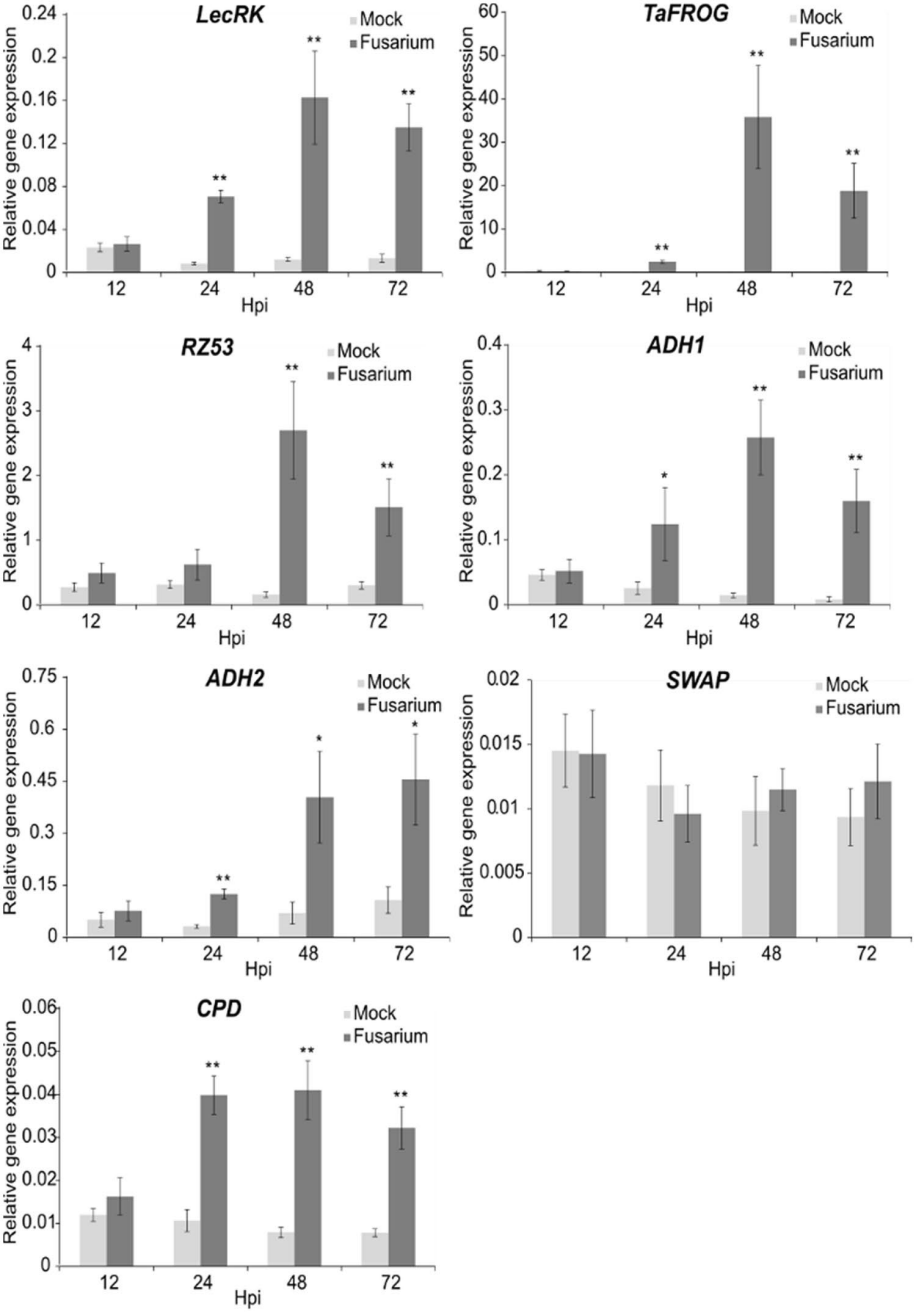
Expression profiles of *Fusarium*-responsive genes within the TaFROG cluster. Transcript levels in wheat (cv. CM-82036) heads after treatment with *Fusarium graminearum* was measured by qRT-PCR using the formula 2^−(Ct target gene—Ct average housekeeping genes)^. Spikelets were harvested at various hours post-inoculation (hpi) as indicated. Results represent the mean of three independent trials (each include two technical replicates per treatment from a pooled of 4 biological sample) and error bars represent ± S.E.M. Asterisks show significant differences (*P < 0.05, **P < 0.01).

As stipulated by Nutzmann et al.^8^, it is interesting to consider that nonhomologous gene clusters, which are not secondary metabolism clusters, might be present in plant genomes. It is possible that nonhomologous, non-metabolic co-expressed genes may be involved in the same biological process, triggered by *Fusarium* treatment. Six nonhomologous non-metabolic gene clusters (FRGCs 3, 12, 26, 35, 39 & 40) contain genes that are strongly co-expressed (Supplementary Fig. S2, Table S3). One of the characteristics of these clusters is that they all contain at least one receptor-like kinase (RLK) gene (Table 1, Supplementary Table S1). Interestingly, FRGC 26 encompasses the D genome homoeologue of the recently characterised Pooideae-specific *Fusarium* resistance orphan gene (*TaFROG-A*), an orphan gene known to contribute to resistance to *F. graminearum* and the mycotoxin DON^47^. Other *Fusarium*-responsive genes within this cluster include the well-conserved alcohol dehydrogenase (ADH) genes *Adh1* and *Adh2*, the Poaceae-specific uncharacterised gene *RZ53*, a *LecRK*, a SWAP/surp domain-containing protein (*SWAP*) and a carboxypeptidase (*CDP*) gene (Fig. 3d). Three other genes are present but not responsive to *Fusarium*. Genes within this locus are previously shown to be important in the plant stress response. RZ53 was used to delineate QTL for aluminium and cold tolerance, and brown planthopper resistance in rice^48–50^. The closest wheat *LecRK* (L-type lectin receptor kinases) in Arabidopsis, *LecRK-IX.1*, regulates *Phytophthora* resistance and controls cell death in *Nicotiana benthamiana*^51, 52^. *HvADH1*, the barley orthologue of *ADH1,* reduces powdery mildew penetration when silenced and increases fungal penetration success when overexpressed^53, 54^. The carboxypeptidase (*CPD*) gene codes for an orthologue of the Arabidopsis protein SCPL42 (AT5G42240), which belong to the serine carboxypeptidase clade 2^55^. The expression of the genes within FRGC 26 was confirmed by quantitative reverse transcriptase PCR (qRT-PCR) in an independent experiment using the wheat FHB-resistant cultivar CM-82036, with all the genes being induced by *Fusarium* treatment at different time points post-inoculation, except for *SWAP* (Fig. 4). To conclude, it appears that except for *SWAP*, all of the genes within the *TaFROG-D* cluster are directly or indirectly linked to stress resistance and are highly co-expressed upon *Fusarium* treatment. It is known for metabolic gene clusters that co-expression is an indicator of genes functioning in the same biological process^56^. Therefore, we speculate that like *TaFROG-A*, *TaFROG-D* and the other *Fusarium-*responsive genes within this cluster may be involved in a similar biological process and may contribute to *Fusarium* resistance. We note that the *TaFROG-D* cluster genes are conserved on chromosomes A and B, but fewer of them are *Fusarium*-responsive (Supplementary Table S2) and were therefore not detected as significant clusters. We notice in particular that the Poaceae-specific gene *RZ53* is adjacent to, and highly co-expressed with *TaFROG-D* (*r* = 0.99) (Figs. 3d, 4). In addition to *TaFROG-D* and *RZ53*, the cluster also contains a *LecRK*. Interestingly, orphans and poplar-specific genes were found to be frequently co-located with leucine-rich repeat (LRR) domain receptor-like proteins within poplar^57^. Based on expression data, the authors suggested that a pair of physically associated poplar-specific and receptor-like gene products might be functionally linked in response to wounding stress. Similarly, we proposed that the LRR might be functionally linked to Poaceae-specific genes in response to *Fusarium*. Future studies will need to explore potential functional linked between *TaFROG* and the other genes in the cluster, and their impact on *Fusarium* resistance.

### Potential *Fusarium*-responsive gene clusters linked with FHB QTL

As the wheat genotypes used in this study segregate for FHB QTL, we compared gene expression data from FRGCs in NIL38 (carrying QTL *Fhb1* and *Qfhs.ifa-5A*) and NIL51 wheat (which lacks these two QTL). In most of the cases, expression of *Fusarium*-responsive genes was similar across genotypes. 30 of the 44 FRGCs (68%) appeared independently in both NILs, and components of most FRGCs were represented in both NILs (Supplementary Table S4). FRGCs 2, 9, 16 and 33 (on chromosomes 1A, 2A, 2D and 5B, respectively) passed the significance threshold as a cluster in one but not both NILs. Overall, this indicates that the expression of the genes within the FRGCs is not dependent on the presence/absence of the FHB QTL *Fhb1* and *Qfhs.ifa-5A*.

**Figure 5:**
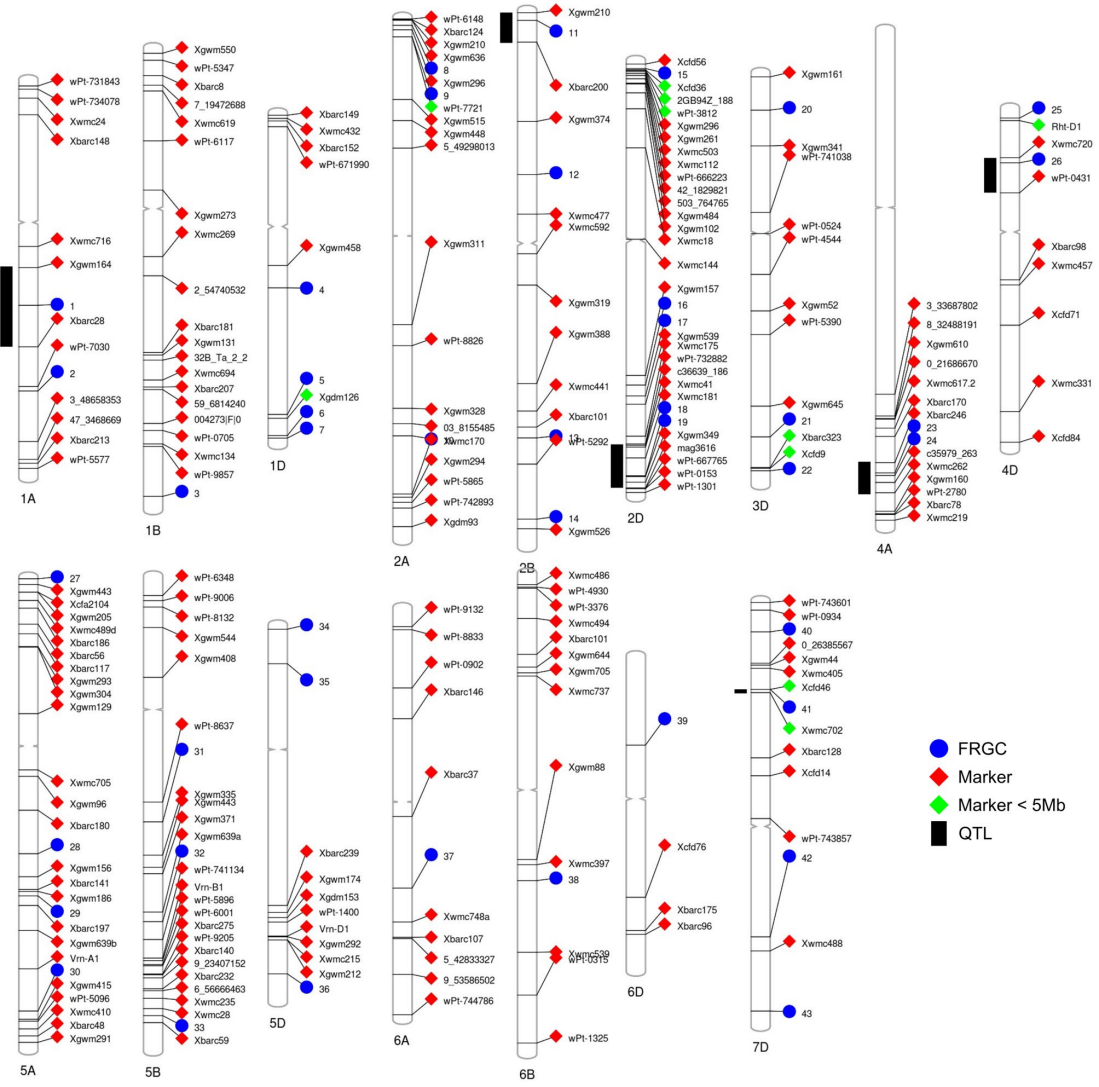
Chromosomal distribution of FRGCs, FHB QTL and associated markers. *Fusarium*-responsive gene clusters and 216 FHB QTL molecular markers were mapped based on their genome positions on the 21 wheat chromosomes. Six QTL regions (Chromosome 1A, 2B, 2D, 4A, 4D and 7D), in which the distance between flanking markers is less than 150 Mb and encompass FRGCs, are indicated with a black vertical bar. FRGCs are represented with a blue circle, and markers with a square, either red if the marker is physically > 5 Mb from a cluster, or green if the marker is physically < 5 Mb from a cluster. Centromeric region are represented as the restricted central region of the chromosomes.

To explore proximity of FRGCs to other known FHB QTL, we physically positioned 216 molecular markers linked to FHB QTL, from a recent compilation of the 556 QTL related to FHB resistance^58^. The physical position of each marker was compared to the position of the 44 FRGCs to identify if any FRGCs are close to FHB QTL (Fig. 5). We found 6 FHB QTL less than 150 Mb wide that each encompass one to two FRGCs (Fig. 5, Table 2). Although 5 of these QTL regions were large (44.2–118 Mb) and contain many genes (472–1071 genes), we noted that QTL-1A, QTL-2B and QTL-4D regions each include one of the FRGCs previously described in this study; a GST cluster (FRGC 1), the benzoxazinoid-like gene cluster (FRGC 11) and the TaFROG gene cluster (FRGC 26), respectively. On chromosome 7D, QTL-7D has the smallest length (4.7 Mb) and contains only 43 high confidence genes based on the wheat reference gene annotation (Supplementary Table S5). Eight of these genes were *Fusarium*-responsive, of which five form the cluster FRGC 41. These genes code for three paralogous EXORDIUM-like 1 proteins, a glycosyltransferase, and an RNA-binding protein. All five genes were induced at 36 and 48 hpi in the FHB resistant NIL-38, whereas in FHB susceptible NIL-51, two EXORDIUM-like 1 and the RNA-binding genes were induced at only 48 h, and one EXORDIUM-like gene was not differentially expressed (Supplementary Table S4). FRGC 41 genes had a weak overall co-expression correlation (*r* = 0.49) but a high co-expression of the three EXORDIUM-like genes (*r* = 0.87) (Supplementary Fig. S2, Table S3). FRGC 41 contains genes potentially linked to FHB resistance mechanisms. Glycosyltransferases belong to the uridine diphosphate (UDP)-glycosyltransferases (UGTs) family, other members of which contribute to DON detoxification and FHB resistance in Poaceae species^59, 60^. The three EXORDIUM-like 1 proteins share 55% identity with the *A. thaliana* EXORDIUM (EXO), a regulator of brassinosteroid (BR)-responsive genes^61^. *EXO* is a gene marker of BR-responses in *A. thaliana* vegetative tissue and, based on mutant studies, it is suggested that EXO contributes to a signalling process modulating BR-responses^62^. Mutation of (BR)-insensitive 1 (*BRI1*), the main receptor of BR, enhanced barley resistance to FHB and Fusarium crown rot (FCR) caused by *F. culmorum*^63, 64^. Additionally, a T-DNA insertion in the 5′ UTR of *Brachypodium distachyon BRI1* impaired BR signalling and reduced susceptibility to FHB and Fusarium root rot (FRR)^63^. Thus, based on small number of genes within the QTL-7D physical region, their *Fusarium* responsiveness, and potential function of genes within the cluster in response to FHB, further studies are worthwhile in order to uncover potential role of FRGC 41 genes in FHB resistance.

**Table 2:** FHB QTL that are smaller than 150 Mb and encompass FRGC.

QTL	Chromosomes	Sources	Flanking markers		Distance between markers (Mb)	Cluster(s) within QTL	References
QTL-1A	1A	Pelikan	Xgwm164	Xbarc28	118	1	Häberle et al.^65^
QTL-2B	2B	Goldfield	Xbarc200	Xgwm210-2B	44.2	11	Gilsinger et al.^66^
QTL-2D	2D	Frontana	wPt-732882	wPt-667765	67.4	18, 19	Szabó-Hevér et al.^67^
QTL-4A	4A	86ISMN_2137	Xbarc246	Xwmc262	44.4	23, 24	McCartney et al.^68^
QTL-4D	4D	86ISMN_2137	Xwmc720	wPt-0431	52.8	26	McCartney et al.^68^
QTL-7D	7D	Haiyanzhong	Xcfd46	Xwmc702	4.7	41	Li et al.^69^

Of all the markers reported by Venske et al.^58^, 10 molecular markers that are either peak or flanking markers of FHB QTL are positioned less than 5 Mb from FRGC genes (Fig. 5, Supplementary Table S6). In total, 5 clusters (FRGC 5, 9, 15, 22 and 25) were found to be less than 5 Mb from QTL markers. Four of these FRGCs are paralogous clusters, with three containing strongly co-expressed genes, such as the MAPKKKs (FRGC 5), RZ53s (FRGC 22) and germin-like protein 1 s (FRGC 25) clusters, and a ribulose bisphosphate carboxylase small chain (RBCS) cluster (FRGC 9), from which a marker is physically positioned within the cluster region. Finally, the momilactone-like cluster on chromosome 2D (FRGC 15) discussed above, is close to three different markers from three studies using three independent resistant sources (Supplementary Table S6). Thus, we suggest that these FRGCs might be considered in future studies as potential regions containing genes underpinning FHB QTL. It is important to note that our method is limited to genome availabilities and gene expression dataset attributes (*e.g*. time points). For example, *PFT,* one of the genes underpinning the *Fhb1* QTL, is *Fusarium*- responsive^21^, but is not present in the wheat cv. Chinese spring reference genome. Therefore, we were unable to detect it in this study.

## Conclusion

In this study, we present a bioinformatics pipeline capable of identifying potentially functionally related gene clusters. This pipeline is adaptable to any eukaryotic genome, and only requires a reference gene annotation and whole genome expression data. Using this pipeline, we identified 44 physical clusters of *F. graminearum* responsive genes within the bread wheat genome. While clusters of functionally related metabolic genes are common through genomes, we found clusters of non-metabolic and nonhomologous genes that are co-expressed under *Fusarium* infection, and therefore may have co-evolved in response to stress. Additionally, we found that many of the gene clusters are physically close to and/or are within known FHB QTL, and therefore this analysis may serve as an additional layer of genetic information when searching for candidate genes underpinning important QTL.

## Materials and methods

### Wheat genome data

Wheat high confidence gene annotations (IWGSC RefSeq v1.1)^17^ and their coordinates (.BED files) were obtained from the IWGSC sequence repository at URGI (https://wheat-urgi.versailles.inra.fr/Seq-Repository/Annotations) using the JBrowse tool (https://wheat-urgi.versailles.inra.fr/Tools/JBrowse). The wheat genome assembly (IWGSC RefSeq v1.0) as a .fasta file and high confidence genes annotation as GFF3 file (IWGSC RefSeq v1.1) were downloaded using the FTP server from Ensembl plant version 44 (https://plants.ensembl.org/Triticum_aestivum/Info/Index). Wheat gene homoeologues, paralogues, and orthologues in *Arabidopsis thaliana*, *Oryza sativa* and *Hordeum vulgare* were identified via Ensembl Plants BioMart tool.

### RNAseq data collection and expression analysis

The gene expression dataset used in this study details RNAseq of FHB treatment of the near isogenic (NIL) wheat lines NIL-38 and NIL-51^24^. Briefly, these NILs are derived from cross between FHB-resistant line CM-82036 and FHB susceptible cultivar Remus, with CM-82036 used as the recurring parent in the backcross. NIL-38 is homozygous for both resistant alleles at the *Fhb1* and *Qhfs.ifa-5A* QTL, and NIL-51 is homozygous for both susceptible alleles at these QTL^24^. A gene count matrix of this RNAseq dataset was downloaded from www.wheat-expression.com^70, 71^ and differential expression analysis was done as per Benbow et al.^72^, where differential expression was conducted per NIL per timepoint.

### Development of a gene cluster and co-expression analysis pipeline

The gene cluster analysis pipeline was writing in the R programming language^73^, and determines density and consecutiveness of disease responsive genes. Gene density is defined as the number of disease responsive genes in the given window (i.e. 5 disease responsive genes in a window size of 10 = a density of 0.5). Consecutiveness is the number of disease responsive genes in a continuous string within a window, i.e. 5 disease responsive genes in a row, with no non-disease responsive genes between them. The pipeline consists of three main elements, (1) a permutation test to determine a significance threshold *λ*, (2) a cluster analysis to determine density and consecutiveness of disease-responsive genes within a sliding window of size χ (user defined, in this study we used a window size of 10 genes) using the pre-determined threshold *λ*, and (3) determining the correlation coefficients of genes within a cluster versus those outside the cluster. The data inputs are: a .BED file of gene coordinates, with columns “Chromosome”, “Start position (bp)”, “End position (bp)”, and “Gene ID”, and an expression data file, in which column 1 is “GeneID”, and subsequent columns are binary designations of 0 = non differentially expressed, and 1 = differentially expressed for each treatment (i.e. *Fusarium graminearum*). The input files used in this study are supplied as supplementary dataset 3. The permutation test takes a user-defined number of permutations *n* (*n* = 10,000, in this study), and creates *n* random datasets, where the number of differentially expressed genes and the genome coordinates of all genes remains constant, but the binary designation of disease-responsiveness is scrambled across the genome. The gene cluster analysis pipeline is then run on all *n* of these random datasets, and the frequency distribution of gene density and consecutiveness are calculated as a percentage. This allows the user to pick *λ* based on the frequency of *λ* occurring by chance. The sliding window analysis takes the .BED file, the expression matrix, and the predetermined *λ* value and the window size *χ* and calculates the average number of disease-responsive genes within the window, the gene density. Subsequently, the number of consecutive DEGs is calculated. The pipeline returns a .csv file per chromosome with the gene density and number of consecutive DEGs appended to it, and a dot plot showing gene density across the chromosomes. The final part of the pipeline is the correlation analysis. Within this, raw expression data (the count matrix of gene expression across all samples) is extracted for all DEGs within each gene cluster, and Pearson’s correlation coefficient (PCC; *r*) is calculated for every pair of genes. This is repeated on the cluster ‘flanking region’ of *y* genes either side of the cluster (*y* is user-defined; *y* = 20 in these study). The average of absolute PCCs is calculated, and a Kruskall-Wallis test is conducted to determine if the absolute PCC within a cluster is significantly different to the absolute PCC outside the cluster. The analysis pipeline scripts are all available at https://github.com/hbenbow/Gene_clusters. All plots created by the analysis pipeline are generated with the R package ‘ggplot2’^74^.

### Gene and metabolic cluster annotation

The wheat gene annotation corresponds to the “Human-Readable-Description” from the IWGSC RefSeq v1.0 annotation. Gene description were updated for the MAP kinase kinase kinase genes utilising homology with *Arabidopsis* MAP kinases, for NLR with the NLR recent annotation^75^, and for the CYP genes using homology and nomenclature of rice CYPs^76^ combined with the wheat CYPs nomenclature curated on the wheat TGACv1 annotation (personal communication by Professor David Nelson, University of Tennessee). Cluster genes were manually classified as metabolic/non-metabolic based on their gene annotation, KEGG pathway ID and Plant reactome ID^77^ retrieved via Ensembl Plants BioMart tool. Additionally secondary metabolic clusters were predicted for each chromosome of the wheat genome with the PlantiSMASH online tool (http://plantismash.secondarymetabolites.org/^29^) using default parameters. PlantiSMASH output results for each chromosome are reported in Supplementary Dataset 1. Finally, clusters were classed as metabolic if ≥ 60% of genes were metabolic or if more than 50% of the genes within the cluster were recognised as secondary metabolic genes by the PlantiSMASH tool.

### Plant material for gene expression

Cultivated plant bread wheat (*Triticum aestivum*) cultivar CM-82036 (kindly provided by Hermann Buerstmayr, BOKU) was used in this study^78^. CM-82036 emerges from the cross Sumai-3/Thornbird-S and was developed by the International Maize and Wheat Improvement Center (CIMMYT, https://www.cimmyt.org). This cultivar can be obtained from the CIMMYT wheat germplasm bank (1.10.6) under the identifier BW 45,200 (plant name CM82036-ITP-10Y-0SJ-10Y-10M-0FC). This cultivar is resistant to FHB and carries two QTL for FHB resistance (*Fhb1* and *Qhfs.ifa-5A*) (as does its’ NIL38 derivative used to generate the RNAseq data analysed in this study). Plant growth conditions were similar to Perochon et al.^79^, and carried out under contained glasshouse conditions. *Fusarium graminearum* wild type strain GZ3639 was used in this study^80^. Fungal mycelium stored at − 80 °C was subcultured on potato dextrose agar (PDA) (Difco, UK) and plates were incubated 5 days at 25 °C. Fungal spores were produced in mung bean broth^81^, harvested, washed and adjusted to 10^6^ conidia ml^-1^ in 0.02% Tween 20, as previously described^82^. At wheat mid-anthesis, two central spikelets of the heads were inoculated with either 20 µl 0.02% (v/v) Tween 20 (mock) or this solution added with 10^6^ conidia of *F. graminearum*. Treated heads were covered with plastic bags for 2 d before being harvested at various timepoints post-inoculation and were flash frozen in liquid N_2_ prior to RNA extraction. The experiment comprised three independent trials, each including eight heads from four individual plants (two heads per plant) per treatment combination. RNA was extracted from one pooled sample per treatment (representing a pool of 8 heads). RNA extractions and cDNA synthesis were performed as described previously by Perochon et al.^79^. Experimental research on plants (glasshouse studies on cultivated wheat, including obtaining the wheat material from BOKU University) complied with relevant UCD, Irish, EU and international guidelines and legislation.

### Quantitative reverse transcriptase PCR analysis

qRT-PCR analysis was conducted using the Mx3000 Real-Time PCR (Stratagene, Germany). Homoeologue-specific PCR primers used in this study (listed in Supplementary Table S7) were designed using Primer3web (http://primer3.ut.ee/^83^) and Genome Specific Primer (GSP) online tools^84^. Specificity of the primers was verified by PCR of DNA extracts from nullisomic-tetrasomic lines of cv. Chinese Spring (obtained from Germplasm Resources Unit, JIC, Norwich http://www.jic.ac.uk/germplasm/), analysis of the dissociation curve, electrophoretic profile of PCR products and sequence analysis. Housekeeping genes used were phosphatase 2A subunit A3 (*TaPP2AA3*) and Yellow-leaf specific gene 8 (*TaYLS8*) and were previously described to be not differentially expressed in FHB experiments^85^. Each qRT-PCR reaction contained 1.25 μL of cDNA, 0.2 μm of each of the primers (except for *LecRK* and *CPD*, concentrations were 0.15 and 0.1 μm, respectively) and 1 × SYBR Premix Ex Taq (Tli RNase H plus, RR420A; Takara) in a total reaction volume of 12.5 μL. PCR conditions were as follows: 1 cycle of 1 min at 95 °C; 40 cycles of 5 s at 95 °C and 20 s at 60 °C; and a final cycle of 1 min at 95 °C, 30 s, at 55 °C, and 30 s at 95 °C for the dissociation curve. All qPCR analyses were conducted in duplicate using two cDNA generated from independent reverse transcriptions. The threshold cycle (Ct) values obtained by qPCR were used to calculate the relative gene expression using the formula 2^−(Ct target gene – Ct housekeeping gene)^ as described previously^86^.

### Identification of FRGCs that lie within FHB QTL intervals

To identify if any of the FRGCs were physically located within a previously identified QTL region for *Fusarium* resistance, we used wheat FHB QTL information documented by a systematic review of the wheat FHB QTLome^58^. We extracted marker information linked to QTL and downloaded their physically positions from wheat JBrowse (https://wheat-urgi.versailles.inra.fr/Tools/JBrowse). Using a bespoke R script (available at https://github.com/hbenbow/Gene_clusters), we retrieved the left and right position (cM) of each QTL. The physical position of each of these markers (in bp) was extracted, and all markers for which the chromosome designation for the physical map and the genetic map did not agree were removed. A subset of the genome .BED file was then extracted, retrieving only the coordinates within the physical QTL interval. For each QTL interval, FRGCs were identified within the region, and the physical size of the QTL region was identified. For QTL where information on flanking markers were reported, we identified their physical size, chromosomal position and if they contained FRGCs. For every marker we calculated their distance from a FRGC. The figure of the chromosomal distribution of QTL, markers and FRGCs was generated using the visualisation tool from Ritchie lab (http://visualization.ritchielab.org/phenograms/plot).

### Statistical analysis and figure drawing 

The normality of the data distribution was evaluated with the Shapiro–Wilk test using R^73^ and SPSS statistics version 24 software (IBM). Statistical analyses were performed with R for cluster gene co-expression using Kruskall-Wallis, with SPSS for qRT-PCR gene expression analyses using Mann–Whitney U test and comparing individual treatments. All figures were draw using the free vector graphics software Inkscape 1.0 (https://inkscape.org/).

## Supplementary Information

Supplementary Figure S1

Supplementary Figure S2

Supplementary Dataset 1

Supplementary Dataset 2

Supplementary Dataset 3

Supplementary Information

Supplementary Table S1

Supplementary Table S2

Supplementary Table S3

Supplementary Table S4

Supplementary Table S5

Supplementary Table S6

Supplementary Table S7

## Data Availability

All data generated or analysed during this study are included in this published article (and its Supplementary Information files). Datasets are also available from the corresponding author on reasonable request and the wheat cultivar used in this study can be obtained from the CIMMYT wheat germplasm bank (1.10.6) under the identifier BW 45,200 (plant name CM82036-ITP-10Y-0SJ-10Y-10M-0FC).
