# Supplementary material for: Analysis of the chromosomal clustering of Fusarium-responsive wheat genes uncovers new players in the defence against head blight disease
Source: Sci Rep. 2021 Apr 2;11:7446. doi: 10.1038/s41598-021-86362-4 (PMC8018971; doi:10.1038/s41598-021-86362-4)
Supplement: Supplementary file 1 — Supplementary Figure S1 [file 41598_2021_86362_MOESM1_ESM.pdf]

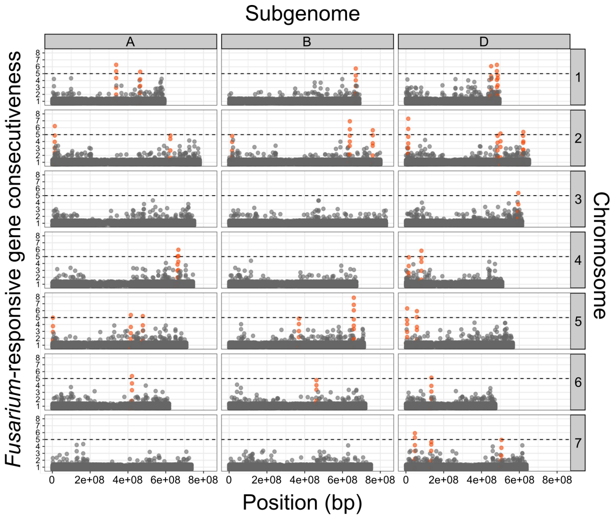

Figure S1. The consecutiveness of *Fusarium*-responsive genes along each wheat chromosome. A sliding window of 10 genes was used to screen each chromosome and calculate the *Fusarium*-responsive gene consecutiveness. A significance threshold (dash line) was calculated by 1000 random permutations of the data and enriched *Fusarium*-responsive gene loci (those that passed the threshold) were identified and are represented in red vermillion.
