# Supplementary material for: Analysis of the chromosomal clustering of Fusarium-responsive wheat genes uncovers new players in the defence against head blight disease
Source: Sci Rep. 2021 Apr 2;11:7446. doi: 10.1038/s41598-021-86362-4 (PMC8018971; doi:10.1038/s41598-021-86362-4)
Supplement: Supplementary file 2 — Supplementary Figure S2 [file 41598_2021_86362_MOESM2_ESM.pdf]

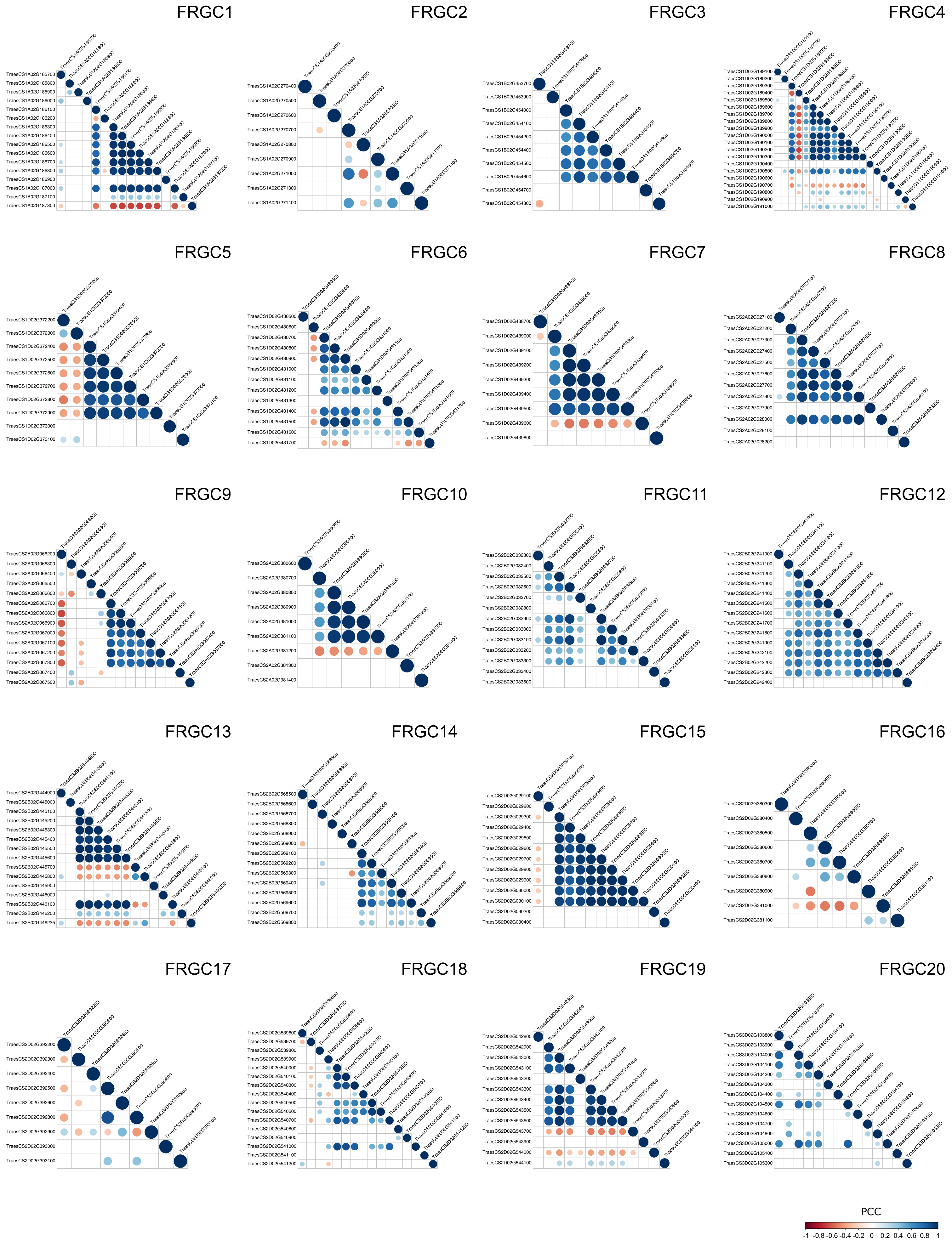
FRGC1
FRGC2
FRGC3
FRGC4
FRGC5
FRGC6
FRGC7
FRGC8
FRGC9
FRGC10
FRGC11
FRGC12
FRGC13
FRGC14
FRGC15
FRGC16
FRGC17
FRGC18
FRGC19
FRGC20
PCC
-1 -0.8 -0.6 -0.4 -0.2 0 0.2 0.4 0.6 0.8 1

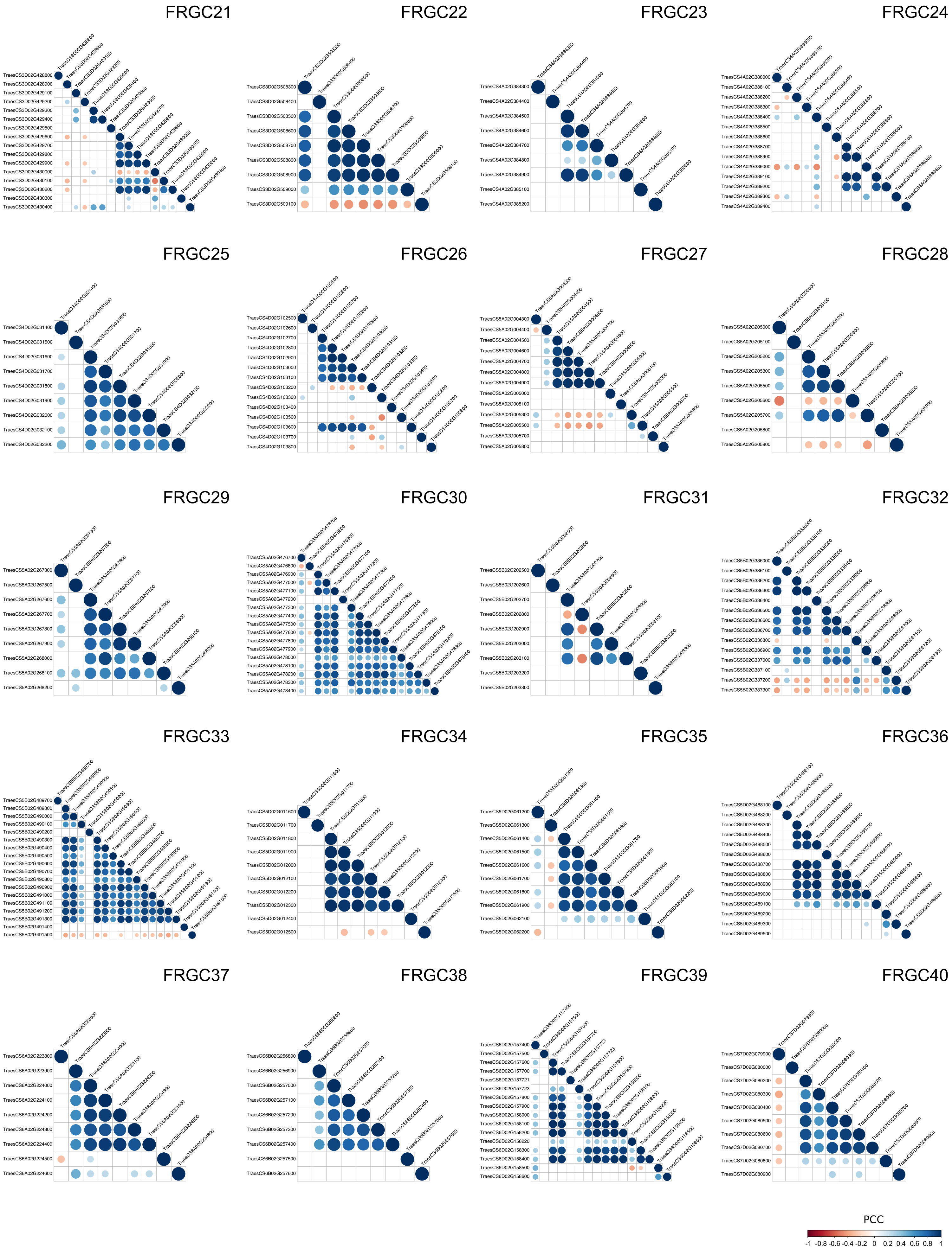
FRGC21
FRGC22
FRGC23
FRGC24
FRGC25
FRGC26
FRGC27
FRGC28
FRGC29
FRGC30
FRGC31
FRGC32
FRGC33
FRGC34
FRGC35
FRGC36
FRGC37
FRGC38
FRGC39
FRGC40
PCC
-1 -0.8 -0.6 -0.4 -0.2 0 0.2 0.4 0.6 0.8 1

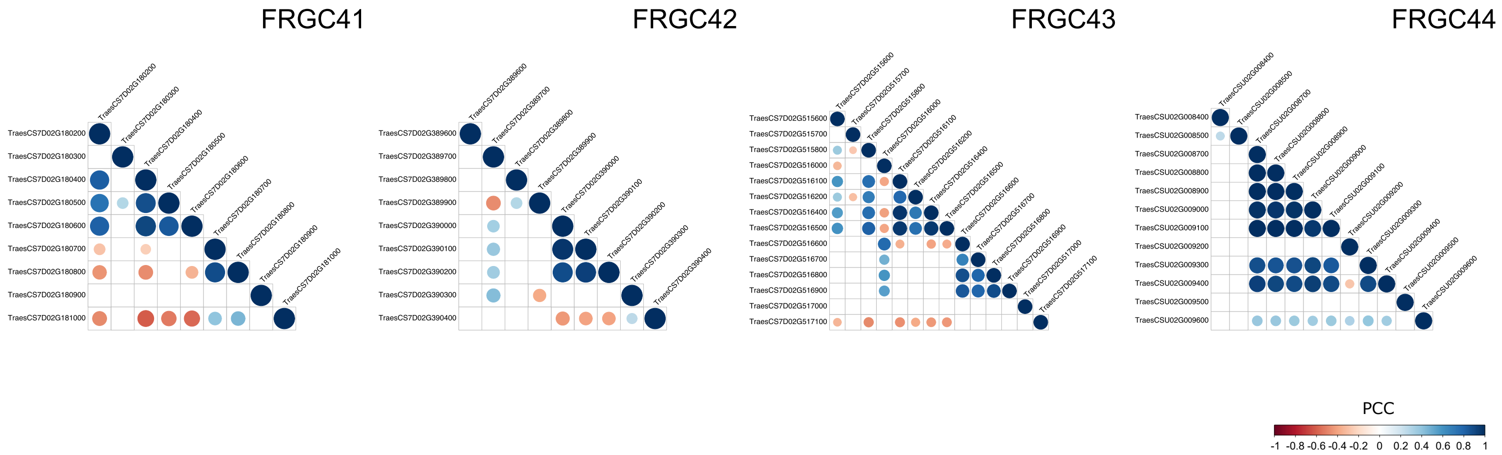

Figure S2. Co-expression matrix of FRGC. Expression of clustered genes plus the two neighbouring genes flanking each side of the cluster were used to calculate to generate a co-expression matrix for each FRGC. Circles characteristics represent the direction (colour, blue = positive, red = negative) and the strength (size) of the correlation.
