# Supplementary material for: Analysis of the chromosomal clustering of Fusarium-responsive wheat genes uncovers new players in the defence against head blight disease
Source: Sci Rep. 2021 Apr 2;11:7446. doi: 10.1038/s41598-021-86362-4 (PMC8018971; doi:10.1038/s41598-021-86362-4)
Supplement: Supplementary file 6 — Supplementary Information [file 41598_2021_86362_MOESM6_ESM.docx]

**SUPPLEMENTARY INFORMATION**

Analysis of the chromosomal clustering of *Fusarium-*responsive wheat genes uncovers new players in the defence against head blight disease

Alexandre Perochon^1,+^, Harriet R. Benbow^1,+^, Katarzyna Ślęczka-Brady^1^, Keshav B. Malla^1^ and Fiona M. Doohan^1,^*

^1^UCD School of Biology and Environmental Science and Earth Institute, College of Science, University College Dublin, Belfield, Dublin 4, Ireland

*corresponding author email: [fiona.doohan@ucd.ie](mailto:fiona.doohan@ucd.ie)

^+^These authors contributed equally to this work

**Supplementary Figure S1.** The consecutiveness of *Fusarium*-responsive genes along each wheat chromosome. A sliding window of 10 genes was used to screen each chromosome and calculate the *Fusarium-*responsive gene consecutiveness. A significance threshold (dash line) was calculated by 1000 random permutations of the data and enriched *Fusarium*-responsive gene loci (those that passed the threshold) were identified and are represented in red vermillion.

**Supplementary Figure S2.** Co-expression matrix of FRGC. Expression of clustered genes plus the two neighbouring genes flanking each side of the cluster were used to calculate to generate a co-expression matrix for each FRGC. Circles characteristics represent the direction (colour, blue = positive, red = negative) and the strength (size) of the correlation.

**Supplementary Dataset 1.** Metabolic gene clusters identified within the wheat genome using PlantiSMASH.

**Supplementary Dataset 2.** Details of metabolic gene cluster identified with PlantiSMASH and matching with FRGC.

**Supplementary Dataset 3.** Input files used to identify FRGC in this study.

**Supplementary Table S1.** Details of *Fusarium*-responsive gene clusters.

**Supplementary Table S2.** Details of *Fusarium*-responsive gene cluster homoeologous regions.

**Supplementary Table S3.** *Fusarium*-responsive gene cluster coexpression correlations.

**Supplementary Table S4.** Expression of FRGC genes in response to *Fusarium graminearum*.

**Supplementary Table S5.** QTL-7D gene annotation and *Fusarium* response.

**Supplementary Table S6.** List of Fusarium-responsive gene clusters positioned less than 5 Mb from a FHB QTL molecular markers.

**Supplementary Table S7.** qRT-PCR primer sets used in this study.
